# Outbreak of *Streptococcus pyogenes emm89* ST646 in a head and neck surgical oncology ward

**DOI:** 10.1128/spectrum.04260-23

**Published:** 2024-04-08

**Authors:** Brian Hayama, Sohei Harada, Masahiro Suzuki, Yohei Doi, Yusuke Nomura, Kotaro Aoki, Kazumi Takehana, Tomomi Akatsuchi, Taisuke Enokida, Koichi Takeda, Akira Seto, Hiroki Mitani, Daisuke Ohkushi

**Affiliations:** 1 Department of Infectious Diseases, Cancer Institute Hospital, Japanese Foundation for Cancer Research, Tokyo, Japan; 2 Department of Infection Prevention, Cancer Institute Hospital, Japanese Foundation for Cancer Research, Tokyo, Japan; 3 Department of Microbiology and Infectious Diseases, Toho University School of Medicine, Tokyo, Japan; 4 Department of Microbiology, Fujita Health University School of Medicine, Toyoake, Aichi, Japan; 5 Department of Infection Control and Prevention, The University of Tokyo Hospital, Tokyo, Japan; 6 Clinical Laboratories, Cancer Institute Hospital, Japanese Foundation for Cancer Research, Tokyo, Japan; 7 Department of Head and Neck Surgery, Cancer Institute Hospital, Japanese Foundation for Cancer Research, Tokyo, Japan; NHLS Tygerberg/Stellenbosch University, Cape Town, Western Cape, South Africa

**Keywords:** *Streptococcus pyogenes*, *emm89*, ST646, outbreak

## Abstract

*Streptococcus pyogenes* causes a variety of human infections, and hospital outbreaks with this pathogen have also been reported. The purpose of this study is to describe the clinical characteristics of an outbreak of *S. pyogenes* involving 15 patients and four healthcare workers (HCWs), as well as the molecular characteristics of the causative isolates. The course and response to the outbreak were reviewed, and information on the characteristics of the patients was extracted retrospectively from the medical records. Whole-genome sequencing of the 16 causative isolates (14 from patients and two from HCWs) was also performed. All 15 patients were postoperative of head and neck cancer with tracheotomy, and 12 had invasive infections, primarily surgical site infections, all of which resolved without causing serious illness. All but the first case was detected more than 7 days after admission. *S. pyogenes* was detected in two patients after empiric antimicrobial administration was performed on all inpatients and HCWs, and the outbreak was finally contained in approximately 2 months. All isolates detected in patients and HCWs belonged to *emm89*/clade 3, a hypervirulent clone that has emerged worldwide and was classified as sequence type 646. These isolates had single nucleotide polymorphism (SNP) differences of zero to one, indicating clonal transmission. This study demonstrated an outbreak of *S. pyogenes emm89*/clade 3 in a ward of patients with head and neck cancer. The global emergence of hypervirulent isolates may increase the risk of outbreaks among high-risk patients.

**IMPORTANCE:**

This study describes an outbreak of *Streptococcus pyogenes* that occurred in a ward caring for patients with head and neck cancer and tracheostomies. Many cases of invasive infections occurred in a short period, and extensive empiric antimicrobial administration on patients and healthcare workers was performed to control the outbreak. Whole-genome sequencing analysis of the causative strains confirmed that it was a monoclonal transmission of strains belonging to *emm89*/clade 3. The epidemiology and clinical characteristics of *S. pyogenes* infections have changed with the replacement of the prevalent clones worldwide. In the 1980s, there was a reemergence of *S. pyogenes* infections in high-income countries due to the spread of hypervirulent emm1 strains. *emm89*/clade 3 has recently been spreading worldwide and shares common features with emm1, including increased production of two toxins, NADase, and streptolysin O. The outbreak reported here may reflect the high spreading potential and virulence of *emm89*/clade 3.

## INTRODUCTION

*Streptococcus pyogenes* is a β-hemolytic Gram-positive coccus that is responsible for more than 500,000 deaths per year worldwide (1). It can cause a variety of diseases that vary from mild pharyngitis to severe invasive infections, which is a mixture of several conditions, such as necrotizing fasciitis, streptococcal toxic shock syndrome (STSS), or sepsis with or without bacteremia. Mortality from *S. pyogenes* infections now occurs mainly in low- and middle-income countries, where malnutrition, poor sanitation, and overpopulation exist (1). *S. pyogenes* infections had diminished in high-income countries from around 1950 likely owing to the amelioration of the factors listed above. However, it reemerged in developed countries in the 1980s and has become a major health problem again (2). This reemergence is attributed to the development of a pandemic type, *emm1*, which has acquired hypervirulent traits through several horizontal gene transfer events (3, 4).

*emm* typing is a method to genetically classify *S. pyogenes* based on the homology of genes encoding the M protein, the most important virulence factor, and more than 200 *emm* types have been reported thus far (5). The global distribution of *emm* types has changed over time. Although isolates of the same *emm* type share common genetic characteristics and are sometimes associated with specific diseases, genetic diversity can occur even among isolates of the same *emm* type. Invasive infections caused by *emm89* isolates have been increasingly reported worldwide in recent years (6–9). A recent meta-analysis of the global distribution of *emm* types among *S. pyogenes* clinical isolates showed that *emm89* is one of the most frequently detected *emm* types in high-income countries, along with *emm1*, *emm12*, *emm28*, etc. (10). Interestingly, the increase in *emm89* has been accompanied by the emergence and rapid domination of a novel clade within *emm89*, clade 3.

A similar situation was observed in Japan, where genetic analysis of *S. pyogenes* isolates associated with STSS isolated in 2013–2018 showed that *emm89* accounted for 23.3% of all isolates and was the second most common type after *emm1* (11). In addition, another study showed that 156 (96.9%) of 161 *emm89* clinical isolates from 2011 to 2019 in Japan belonged to clade 3 (12). During the coronavirus disease 2019 (COVID-19) pandemic, when the number of STSS cases in Japan was reduced, the proportion of *emm89* strains remained stable, despite a decrease in the proportion of *emm1* strains among the causative strains (13).

In this study, we describe an outbreak in a hospital ward involving postoperative patients with head and neck cancer and healthcare workers and present the results of genetic analysis supporting the clonal spread of *emm89*/clade 3 isolates.

## MATERIALS AND METHODS

### Setting and design

This was a retrospective descriptive study of the clinical and molecular characteristics of an outbreak of *S. pyogenes* in the head and neck surgery ward of a 686-bed cancer hospital. The head and neck surgery ward consists of 10 single-patient rooms and nine four-patient rooms, and more than 90% of the patients admitted are diagnosed with head and neck cancer. Nearly all patients admitted to the hospital for elective surgery for head and neck cancer are admitted to this ward, and most postoperative outpatients are also admitted to this ward unless the main purpose of admission is cancer chemotherapy. Inpatients of the ward who have recently undergone surgery and/or have tracheotomy receive wound and airway care from physicians every morning sequentially in the procedure room of the ward, which has two treatment units accompanied by tables equipped with instruments and disposable items for the procedure. The case definition of patients in this study was inpatients of the ward with *S. pyogenes* detected in culture tests from April through June 2019. The case definition of healthcare workers (HCWs) in this study was HCWs dedicated to the head and neck surgery ward (non-physicians) or department (physicians) who had *S. pyogenes* detected in culture tests or were positive for group A streptococci in rapid antigen tests of throat swabs in the same period.

Isolation of bacteria from blood samples was performed using the BACT/ALERT 3D system (bioMérieux Japan Ltd., Tokyo, Japan). Isolation of *S. pyogenes* from clinical specimens was performed using horse blood agar medium (Kyokuto Pharmaceutical Industrial Co., Ltd., Tokyo, Japan). Identification of *S. pyogenes* in the microbiology laboratory of the hospital was performed with MicroScan WalkAway (Beckman Coulter, Brea, CA). Antimicrobial susceptibility testing was performed by disk diffusion methods using Kirby–Bauer disks (Eiken Chemical Co., Ltd., Tokyo, Japan) and the results were interpreted according to the CLSI M100-S27 guidelines (14).

### Clinical data collection

The following clinical information was collected from electronic medical records for patients meeting the case definition: age; gender; type of malignancy diagnosed; date of admission; date of surgery prior to *S. pyogenes* detection; date of collection and specimen type of the culture test from which *S. pyogenes* was detected; wound or airway procedure in the ward procedure room within 7 days prior to *S. pyogenes* detection; administration of antimicrobial agents active against *S. pyogenes* within 7 days prior to *S. pyogenes* detection; the presence of infection due to *S. pyogenes*; diagnosis of *S. pyogenes* infection according to CDC/NHSN criteria (15); cure of *S. pyogenes* infection within 30 days following *S. pyogenes* detection; ICU admission due to *S. pyogenes* infection; mortality within 90 days following *S. pyogenes* detection; and presence of tracheotomy.

### Molecular analysis

Whole-genome sequencing of 14 *S*. *pyogenes* isolates from patients and two *S*. *pyogenes* isolates from HCWs was performed with MiSeq (Illumina, San Diego, CA). Library preparation for MiSeq sequencing was performed with Nextera XT DNA Library Prep Kit (Illumina). Libraries were sequenced on a MiSeq system for 600 cycles (300 bp paired-end reads). Raw reads generated by MiSeq were quality-trimmed with fastp (version 0.23.1) and assembled using SPAdes (version 3.13.1).

Multilocus sequence typing (MLST) and *emm* typing with draft genome data of the isolates were performed on the pubMLST website (https://pubmlst.org/organisms/streptococcus-pyogenes). In addition, core-genome single nucleotide polymorphism (SNP) analysis was performed. The MiSeq sequencing reads were aligned to the genomic sequence of the reference isolate, *S. pyogenes* MGAS27061 (GenBank accession number: CP013840), using the Burrows–Wheeler Aligner (BWA) (version 0.7.17) with “sw” option (16). Genomic sequence data of *S. pyogenes* MGAS11027 (GenBank accession number: CP013838), MGAS23530 (GenBank accession number: CP013839), and MGAS27061 were added in the analysis as representative isolates of clade 1, clade 2, and clade 3 of *emm89*, respectively. We constructed a core-genome alignment using SAMtools (version 1.17) mpileup (17) and VarScan (version 2.2.9) mpileup2cns (18) and then a maximum-likelihood tree using PhyML (version 3.3) (19).

We performed a phylogenetic analysis of the representative strain of this outbreak (FUJ00398) and the *emm89* strains isolated in Japan that have been reported in previous articles and registered in GenBank. A PubMed search was conducted using the search terms *“emm89*” and “Japan” to identify the relevant publications. Genomic sequencing data of the targeted strains was obtained from GenBank (accession number: DRA009110 and AP017629). Genomic sequence reads of the target strains and MGAS11027, MGAS23530, and MGAS27061 was aligned to the genomic sequence of the reference strain (MGAS23530) using the BWA with the “sw” option. We obtained the provisional core-genome sequences including homologous recombination sites and SNPs using SAMtools mpileup and VarScan mpileup2cns, both with default parameters. A phylogenetic tree of the provisional core-genome sequences of the strains was estimated using PhyML. Using this as the starting tree, we inferred homologous recombination sites, which should be excluded from the core genomes, using ClonalFrameML (20). SNPs were determined by excluding SNPs that were present in the homologous recombination sites from SNPs that were present in the provisional core genome. The phylogenetic tree based on SNPs present in the core-genome excluding homologous recombination sites was estimated by maximum likelihood estimation using axmlHPC-PTHREADS command and GTRGAMMA model of RAxML (version 8.2.12) (21).

## RESULTS

### Outbreak description

*S. pyogenes* was detected from blood cultures of two inpatients of the ward taken on 9 April 2019 (Table 1). In response, the results of the microbiology tests of all inpatients of the ward in the previous month were reviewed by the infection control team, and the detection of *S. pyogenes* from culture specimens of two other patients submitted on 3 April and 6 April was recognized. Subsequently, *S. pyogenes* was detected from culture specimens collected from three other patients in the same ward on 11, 14, and 16 April, which led to the initiation of the intervention by the hospital infection control team. Ward HCWs were educated by the infection control team on adherence to hand hygiene and standard precautions. Standard precautions included the use of gloves and gowns during contact with exudate and the use of masks and eye protection during anticipated droplet contamination, but universal masking during work was not required.

**TABLE 1:** Cases with *S. pyogenes* detection during the outbreak^*d*^

Case	Strain^*a*^	Detection date	Sample	Sex	Age^*b*^	Infection caused by *S. pyogenes*	Malignancy	Length of hospital stay until detection	Surgery within 30 days	Tracheostomy	Risk of transmission from patients in the same room^*c*^
Patient-1	Unavailable	3 April	Oral swab	M	40–49	Abscesses around the oral tumor	Pharyngeal cancer	<4 days	No	Permanent	No
Patient-2	FUJ00398	6 April	Sputum	F	70–79	None	Cervical esophageal cancer	>30 days	No	Temporary	No
Patient-3	FUJ00399	9 April	Wound pus/swab blood	M	70–79	Bacteremia Soft tissue infection	Pharyngeal cancer	15–30 days	Yes	Permanent	No
Patient-4	FUJ00400	9 April	Wound pus/swab blood	F	60–69	Bacteremia Soft tissue infection	Pharyngeal cancer	15–30 days	Yes	Permanent	No
Patient-5	FUJ00401	11 April	Sputum	F	70–79	None	Nasal and sinus cancer	>30 days	No	Temporary	No
Patient-6	FUJ00403	14 April	Sputum	F	80–89	None	Oral cancer	>30 days	No	Temporary	Yes
Patient-7	FUJ00404	16 April	Wound pus/swab	F	70–79	Soft tissue infection	Thyroid cancer	>30 days	No	Permanent	No
Patient-8	FUJ00405	18 April	Wound pus/swab	M	40–49	Soft tissue infection	Oral cancer	8–14 days	Yes	Temporary	No
Patient-9	FUJ00406	19 April	Wound pus/swab	F	50–59	Soft tissue infection	Oral cancer	15–30 days	Yes	Temporary	No
Patient-10	FUJ00407	19 April	Wound pus/swab	M	70–79	Soft tissue infection	Thyroid cancer	8–14 days	Yes	Permanent	No
Patient-11	FUJ00409	19 April	Sputum	M	70–79	Pneumonia	Pharyngeal cancer	15–30 days	No	Temporary	No
Patient-12	FUJ00408	21 April	Wound pus/swab	M	70–79	Soft tissue infection	Pharyngeal cancer	8–14 days	Yes	Permanent	Yes
Patient-13	FUJ00410	22 April	Wound pus/swab	M	70–79	Soft tissue infection	Laryngeal cancer	8–14 days	Yes	Permanent	Yes
Patient-14	FUJ00413	17 May	Wound pus/swab	M	60–69	Soft tissue infection	Pharyngeal cancer	8–14 days	Yes	Permanent	No
Patient-15	FUJ00414	30 May	Wound pus/swab	F	70–79	Soft tissue infection	Oral cancer	>30 days	No	Temporary	No
Nurse-1	Unavailable	14 April	Pharyngeal swab	F	20–29	Pharyngitis	NA	NA	NA	No	NA
Nurse-2	Unavailable	20 April	Pharyngeal swab	F	20–29	Pharyngitis	NA	NA	NA	No	NA
Nurse-3	FUJ00411	22 April	Pharyngeal swab	F	60–69	Pharyngitis	NA	NA	NA	No	NA
Nurse-4	FUJ00412	23 April	Pharyngeal swab	F	20–29	Pharyngitis	NA	NA	NA	No	NA

^*a*^ Strains were unavailable for one patient (Patient-1) because the strain was not stored and for two nurses (Nurse-1, -2) because they were diagnosed by rapid antigen testing.

^*b*^ Ages are shown in 10-year groups.

^*c*^ The risk of transmission from patients in the same room was considered positive if a patient in the same room had shedding of *S. pyogenes* within 2 days of *S. pyogenes* detection. *S. pyogenes* shedding was defined as up to 24 hours after the initiation of effective antimicrobial therapy, provided that *S. pyogenes* carriage was documented by culture.

^*d*^ M, male; F, female; NA, not applicable.

**Fig 1:**
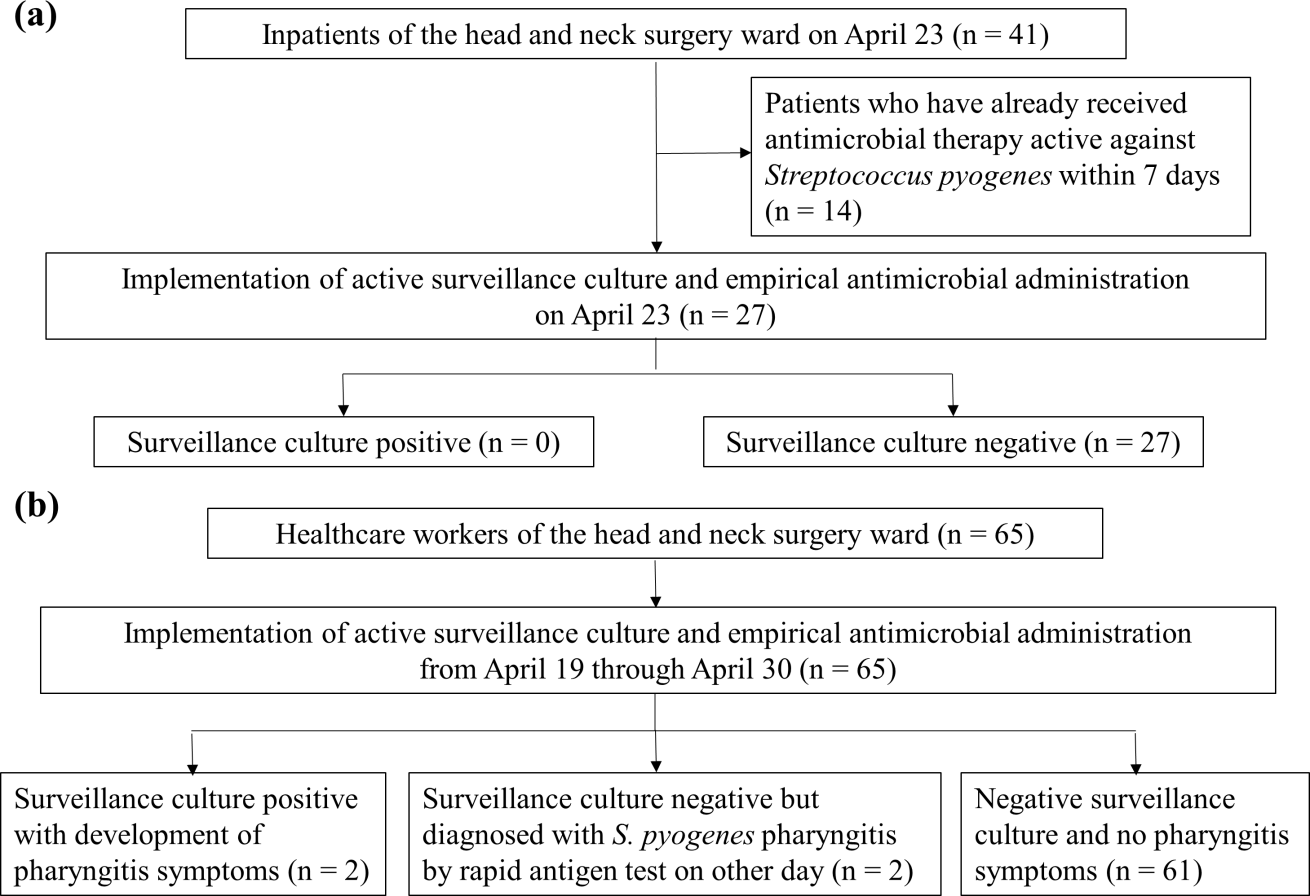
Active surveillance cultures and empirical antimicrobial administration performed on (**a**) hospitalized patients and (**b**) healthcare workers in response to the outbreak.

Due to the subsequent increase in cases of *S. pyogenes* infection, active surveillance culture of pharyngeal swabs was performed and empiric antimicrobial administration was initiated on 23 April for all inpatients in the ward except for those who have received antimicrobial treatment active against *S. pyogenes* within 7 days (Fig. 1a). Because of the sharp increase in cases, empirical antimicrobials were administered without waiting for the results of the culture tests. The antimicrobial agent empirically administered was cephalexin 1,000 mg/day orally for 10 days, and one patient with a history of β-lactam allergy received azithromycin 500 mg/day for 3 days as an alternative agent. Due to the limited supply of antimicrobials to the hospital during this period, cephalexin, which was available in the necessary doses, was used as an alternative to penicillin. Active surveillance culture of pharyngeal swabs and empiric antimicrobial administration was also performed for all 65 ward HCWs including 17 physicians and 34 nurses from 19 April through 30 April (Fig. 1b). As with the patients, cephalexin was prescribed as the empiric antimicrobial, and two HCWs with a history of β-lactam allergy received azithromycin. It was also confirmed that no HCWs had skin lesions suspicious of skin and soft tissue infection. Environmental cultures were collected from patient chairs, handrails, headrests, treatment tables, and light handles in the procedure room, and repeated environmental disinfection of the patient rooms and the procedure room was implemented with alcohol-containing wipes.

**Fig 2:**
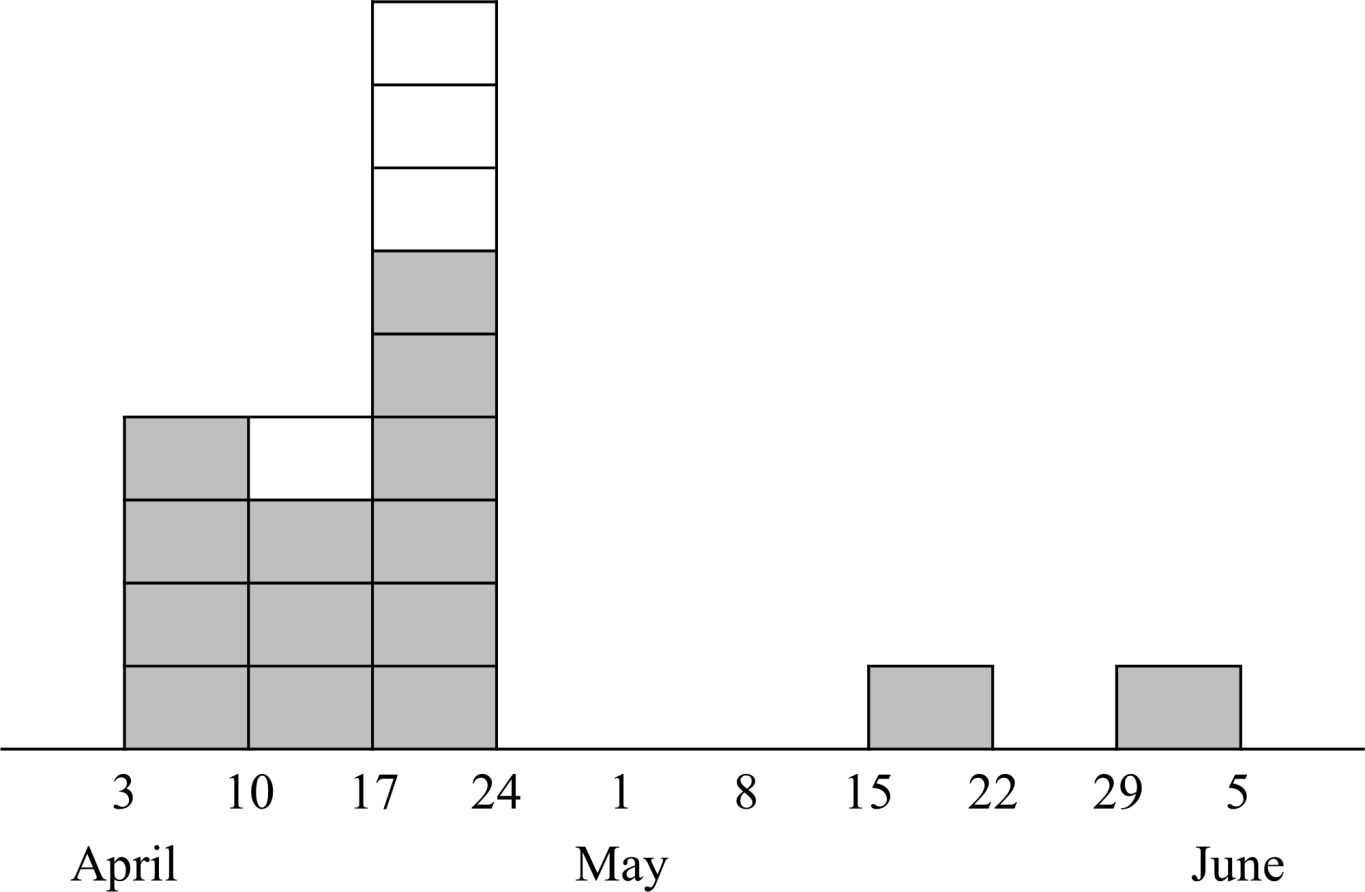
Epidemic curve of *S. pyogenes* detection in the ward. Boxes indicate periods when *S. pyogenes* was detected. Gray and white boxes represent patients and healthcare workers in whom *S. pyogenes* was detected, respectively.

Finally, a total of 15 inpatients were identified with *S. pyogenes* between 3 April and 30 May in the ward (Table 1). All strains were susceptible to penicillin, ampicillin, erythromycin, and levofloxacin. All *S. pyogenes* isolates from the patients were detected in clinical culture specimens submitted at the discretion of the treating clinicians, and none were detected through active surveillance cultures performed as a part of the outbreak response (Fig. 1a). Detection of *S. pyogenes* from patients peaked during the third week of the outbreak, and new detections temporarily ceased after implementation of extensive empiric antimicrobial administration for patients and HCWs, but there were new detections in clinical culture specimens from one patient each on 17 May and 30 May (Fig. 2). One of these two patients was admitted after above-mentioned empiric antimicrobial administration for inpatients and was on his 12th day of hospitalization, and the other patient was more than 30 days post-admission and had been negative for active surveillance culture and had received empiric antimicrobial administration. These incidences suggested the persistence of transmission even after extensive empiric antimicrobial administration. During this period, there were no cases of *S. pyogenes* detection from patients in the ward other than those with head and neck cancer. Of the 65 HCWs for whom surveillance cultures were taken, two nurses (3.1%) tested positive in the third week (Fig. 1 and 2). Although they were identified by active surveillance culture, they had symptoms of pharyngitis at the time the test results arrived. Another two nurses had negative surveillance cultures but positive rapid antigen tests for *S. pyogenes* at clinics where they had visited with pharyngitis symptoms on other days (Table 1; Fig. 1). All four nurses improved quickly after the administration of antimicrobial agents, and they were suspended from work until the administration of antimicrobial agents was completed and their symptoms had improved. Environmental cultures collected from multiple locations in the procedure room were negative for *S. pyogenes*.

The first *S. pyogenes* detection from an inpatient of the ward after 30 May was on 26 August. The number of *S. pyogenes* detected from inpatients of the ward in the 6 months prior to the outbreak (from October 2018 through March 2019) and in the 6 months following the outbreak (from June through November 2019) was one and two, respectively.

### Characteristics of the patients

All 15 patients with *S. pyogenes* detection had head and neck cancer (Table 1). The mean age was 68.4 (standard deviation: 10.4) years and eight patients were male. All patients had temporary or permanent tracheostomy. Although all but one patient (Patient-11) was in a four-patient room on the day of detection, only three patients were in the same room within 2 days prior to detection with a patient carrying *S. pyogenes* before or within 24 hours after the initiation of effective antimicrobial therapy. All patients were detected after more than 7 days of admission, except one patient (Patient-1), who was detected 3 days after admission and was the first patient identified in this outbreak. All patients had a history of procedure room use within 7 days prior to detection of *S. pyogenes*. There were eight patients with a history of surgery within 30 days, all of whom developed skin and soft tissue infections at the surgical site caused by *S. pyogenes*. In addition, two patients had skin and soft tissue infections from surgical wounds of earlier operations, one patient had an abscess at the site of an oral tumor, and one patient had pneumonia. All patients with *S. pyogenes* infection were successfully treated. Of the patients in whom *S. pyogenes* was detected in culture tests, only three patients were asymptomatic. There were no ICU admissions within 7 days or all-cause deaths within 90 days.

### Molecular characteristics of the outbreak isolate

The *S. pyogenes* isolates from 14 patients and two HCWs were available for whole-genome sequencing analysis. Whole-genome sequencing revealed that all these isolates belonged to *emm89* and ST646. SNP analysis was performed on the core genome (95.4% of the 1,741,348 bp genomic sequence of MGAS27061) of these isolates and representative isolates of *emm89* clade 1, 2, and 3. No SNP differences were found among the isolates detected from patients and HCWs in April, and the two isolates detected from patients in May differed by only a single SNP from those detected in April. The SNP was located in the *pyrH* gene (locus_tag: MGAS27061_RS01950). The SNP difference between the representative isolate of the outbreak isolates (FUJ00398) and the representative isolates of clade 1, 2, and 3 was 1,868, 374, and 42, respectively, suggesting that the outbreak isolates belong to clade 3. FUJ00398 had the *emm89*/clade 3-specific promoter sequence of *nga-ifs-slo*, genes for *S. pyogenes* NADase (SPN), an endogenous inhibitor of SPN, and streptolysin O (SLO), respectively, and did not carry the *hasABC*, which is involved in the synthesis of the hyaluronic acid capsule (22).

**Fig 3:**
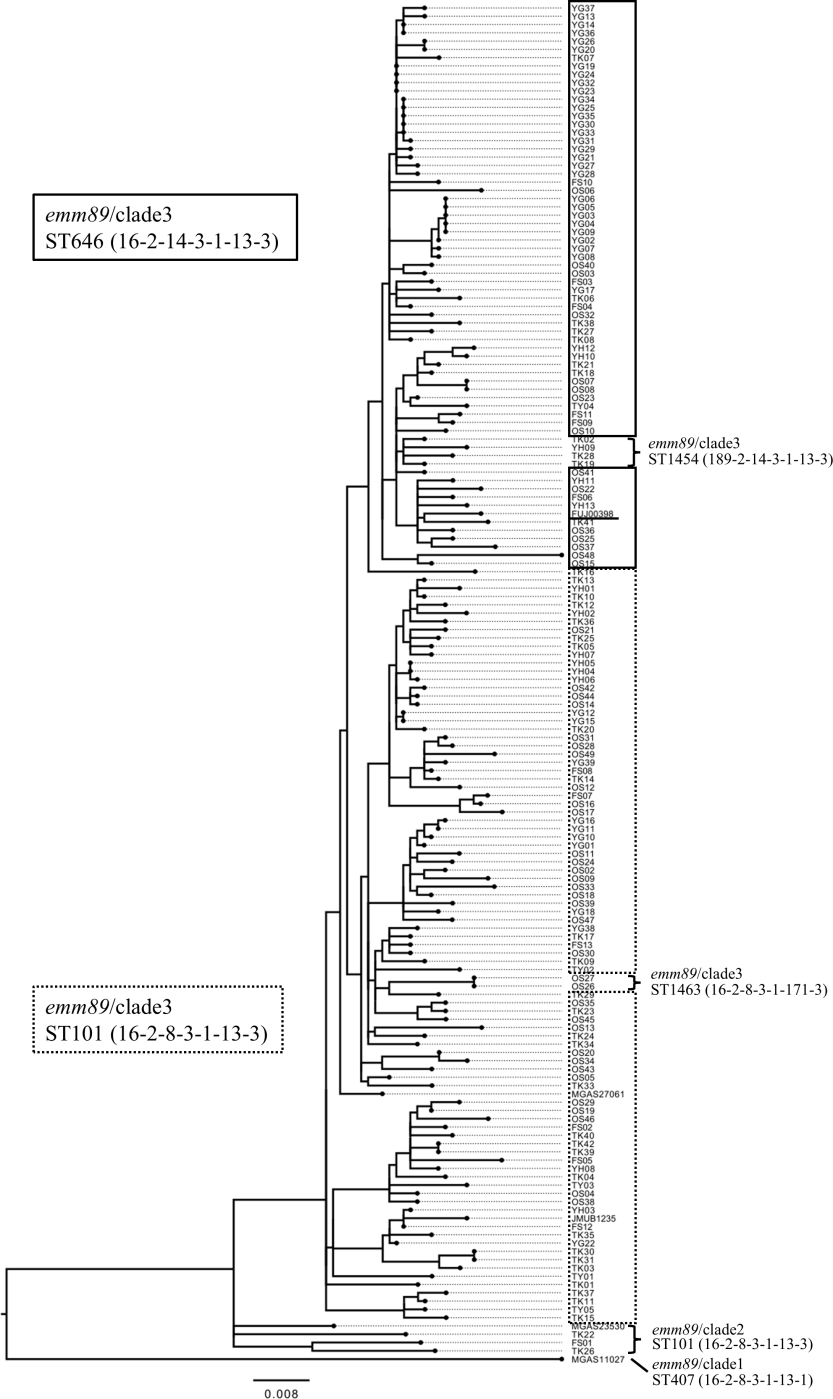
Phylogenetic tree of *emm89* strains isolated in Japan. Allele profile (*gki-gtr-murI-mutS-recP-xpt-yqiL*) for each ST is indicated in parentheses. The representative strain of this outbreak (FUJ00398) is underlined. ST, sequence type.

It was found that genomic sequences of 162 *emm89* strains isolated in Japan were available for comparison by referring to the articles identified by a PubMed search performed on 20 February 2024. All but one strain was derived from a single study (12, 23). The initial construction of a phylogenetic tree identified two genetically highly distant strains (OS01 and TK32), a result that was consistent with the previous publication (12). The final phylogenetic tree was created excluding these two strains to allow for the detection of minor genetic differences. The core genome used for phylogenetic analysis was 1,019,443 bp (59.6% of the 1,709,394 bp genomic sequence of the reference strain MGAS23530). Sixty-four ST646 strains, including the representative strain of this outbreak (FUJ00398), diverged from 93 ST101 strains to form a closely related population (Fig. 3). Four of the ST646 descendant strains were further accompanied by a single nucleotide mutation in the *gki* gene (ST1454).

## DISCUSSION

An outbreak of *S. pyogenes* involving 15 patients and four HCWs occurred in a surgical ward for head and neck cancer patients over a 2-month period, and whole-genome sequencing analysis of the isolates confirmed clonal spread of *emm89*/clade 3 isolates. Twelve patients developed invasive infections, mainly skin and soft tissue infections at the surgical site, but all were cured without critical illness. All four HCWs presented with mild pharyngitis.

Numerous hospital outbreaks of *S. pyogenes* have been reported, occurring in ICUs, medical and surgical wards, and obstetric wards (24). Outbreaks are caused by patient-to-patient transmission, transmission from an HCW carrying the organism, transmission through a contaminated hospital environment, or a combination of the above. HCWs are reported to be more likely to be involved in transmission in outbreaks in surgical or obstetric wards (24). Although the response to hospital outbreaks of *S. pyogenes* has not been fully standardized, the UK guidelines suggest the formation of an outbreak control team, epidemiological investigation including retrospective analysis of hospital-onset *S. pyogenes* infections in the past 6 months, reaffirmation of infection control policies with prompt correction of any flaws, consideration of screening for HCWs and decolonization of positive individuals, identification of common sources of infection (baths, showers, etc.) and subsequent environmental sampling, screening of patients, and chemoprophylaxis (25). In the present case of a ward-wide outbreak, the response was led by the infection control team of the hospital, standard precautions and hand hygiene were reinforced, and screening and empiric antimicrobial administration were performed on all inpatients and HCWs of the ward. In addition, environmental cultures were performed in the procedure rooms, but *S. pyogenes* was not identified in this outbreak investigation.

Although the outbreak was contained within approximately 2 months, there were two new cases after the initiation of the intervention, suggesting that transmission had persisted via an unidentified source. In this outbreak response, individuals with *S. pyogenes* isolation who had been treated for at least 24 hours and whose clinical symptoms had improved were not subject to work suspension (HCWs) or isolation in single-patient room and droplet precautions (patients). Although it is generally believed that shedding of *S. pyogenes* stops within 24 hours of initiation of effective treatment, long-term persistent shedding is possible in practice, and therefore, repeated culture testing after eradication therapy and removal of specific precautions after confirmation of disappearance is suggested in the event of outbreaks (25). In this outbreak, culture testing was not repeated after the initial intervention because there were no new cases for more than 2 weeks after the intervention and the two subsequent cases occurred approximately 2 weeks apart.

Patients with head and neck cancer who have undergone airway modification surgery may be at increased risk of acquiring and spreading *S. pyogenes*. Frequent airway care from HCWs may increase the risk of transmission from HCWs, and extensive and persistent droplet spread from the tracheotomy may increase the risk of transmission to surrounding patients and contamination of the hospital environment. In addition, patients with a tracheostomy have difficulty wearing well-fitting masks. In fact, in an outbreak of *S. pyogenes emm1* involving three patients with tracheostomy, *S. pyogenes* was detected from the curtains of the patient rooms and was suspected as a potential source of an outbreak (26). Although environmental disinfection of the patient rooms and the procedure rooms was reinforced in response to this outbreak, the involvement of the hospital environment is unknown because the culture tests were negative. Considering the possible presence of unidentified persistent carriers and the difficulty in preventing the dispersal of droplets from patients with a tracheostomy, universal masking of HCWs in the head and neck surgery ward, which was introduced in this ward after the COVID-19 pandemic, may be a reasonable option.

The outbreak isolates belonged to *emm89*/clade 3 and ST646, and the SNP difference was 0–1, confirming clonal spread. *S. pyogenes emm89*/clade 3 isolates have recently emerged worldwide as one of the major clones of *S. pyogenes* causing invasive infections (6–9). *emm89*/clade 3 isolates emerged in the early 2000s and increased globally in the late 2000s, replacing the previously dominant *emm89*/clade 1 isolates (27). *emm89*/clade 3 isolates differ from *emm89*/clade 1 and *emm89*/clade 2 isolates in the *nga-ifs-slo* promoter sequence and have two SNPs identical to the preceding highly virulent clone, *emm1* isolates, leading to increased production of two toxins, SPN and SLO (22). *emm89*/clade 3 isolates were also characterized as lacking *hasABC*. Although it is assumed that loss of the capsule is associated with decreased virulence, it has been reported that increased production of SPN and SLO results in high virulence even without the capsule in a mouse infection model (28). Genomic data analysis of internationally collected *S. pyogenes* strains revealed that the combination of a promoter sequence with high expression of *nga-ifs-slo* and a mutation in *has* gene was also observed in several other common genotypes, including *emm28* and *emm87*, which may suggest that this combination may confer some selection advantage over having only one of these characteristics (29).

To date, *emm89*/clade 3 has been reported to belong mainly to ST101 worldwide, while the current outbreak isolate was ST646 (6, 7). ST646 is a variant of ST101 with only a single nucleotide mutation of *murI* among the seven housekeeping gene sequences for MLST. Our phylogenetic analysis for *emm89* strains isolated in Japan revealed that ST646 strains diverged from ST101 strains and many clinical strains of this clone have already been isolated. Two of the eight isolates of *S. pyogenes emm89* detected in 2009–2013 in patients with balanoposthitis in Nagoya, Japan, were reported to be ST646 (the remaining isolates were ST101) (30). Future studies on the epidemiology and clinical characteristics of the *emm89*/clade 3 isolate of ST646 in Japan and worldwide are warranted.

This study has several limitations. First, the origin of this outbreak could not be identified. Since *S. pyogenes* was detected in the first identified patient 3 days after admission to the hospital, it was assumed that the patient developed the disease after an incubation period of community-acquired infection. However, we were unable to confirm the clonality of the isolate from this patient with other isolates because it was not stored, therefore, it is possible that spread among the other patients occurred unrelated to the index patient. Although the timeline of detection suggests that the HCWs acquired *S. pyogenes* from the patients under their care, we cannot rule out the possibility that there were unidentified asymptomatic individuals earlier in the outbreak who may have been the source of the outbreak. Second, we may have missed some carriers because we did not screen the vagina and anus of the HCWs, and the wound and tracheotomy-aspirated sputum of the patients in the sites of active surveillance culture specimen collection. In addition, we may have missed persistent carriers because we did not confirm the clearance of bacteria with culture after treatment. These interventions were planned to be implemented if the outbreak persisted or if severe cases occurred, but the outbreak was contained without these events. Third, molecular typing of *S. pyogenes* strains detected before and after the outbreak period was not performed. This may have missed the true onset and termination of the clonal spread of the *S. pyogenes* strains in the ward.

In conclusion, an outbreak of *S. pyogenes emm89*/clade 3 ST646 involving postoperative patients and HCWs in a head and neck surgery ward was documented. The outbreak was successfully contained in approximately 2 months after the reinforcement of standard precautions and hand hygiene, and implementation of ward-wide active surveillance culture and empiric antimicrobial administration of patients and HCWs. The global spread of hypervirulent clones may increase the risk of developing infections due to nosocomial transmission of *S. pyogenes*, and it is necessary to maintain a high level of standard precautions and hand hygiene, especially in wards caring for high-risk populations including patients with head and neck cancer, and to take appropriate early action in case of possible outbreaks.

## Data Availability

All genome sequences have been deposited in the NCBI database under BioProject accession number PRJNA1006847.
